# Decellularized versus cryopreserved pulmonary allografts for right ventricular outflow tract reconstruction during the Ross procedure: a meta-analysis of short- and long-term outcomes

**DOI:** 10.1186/s43044-021-00226-w

**Published:** 2021-11-07

**Authors:** Adham Ahmed, Sarah Ahmed, Kathryn S. Varghese, Dave M. Mathew, Roshan Pandey, Dillon O. Rogando, Stephanie A. Salazar, Peter J. Fusco, Kenneth H. Levy

**Affiliations:** 1 CUNY School of Medicine, 1589 Amsterdam Avenue, New York, NY 10031 USA; 2 New Dorp High School, New York, NY USA; 3 CUNY City College of New York, New York, NY USA

**Keywords:** Aortic valve, Homograft, Allograft, Decellularized, Ross, Ross-Yacoub, Aortic valve replacement

## Abstract

**Background:**

The ideal conduit for repair of the right ventricular outflow tract (RVOT) during the Ross procedure remains unclear and has yet to be fully elucidated. We perform a pairwise meta-analysis to compare the short-term and long-term outcomes of decellularized versus cryopreserved pulmonary allografts for RVOT reconstruction during the Ross procedure.

**Main body:**

After a comprehensive literature search, studies comparing decellularized and cryopreserved allografts for patients undergoing RVOT reconstruction during the Ross procedure were pooled to perform a pairwise meta-analysis using the random-effects model. Primary outcomes were early mortality and follow-up allograft dysfunction. Secondary outcomes were reintervention rates and follow-up endocarditis. A total of 4 studies including 1687 patients undergoing RVOT reconstruction during the Ross procedure were included. A total of 812 patients received a decellularized pulmonary allograft, while 875 received a cryopreserved pulmonary allograft. Compared to cryopreserved allografts, the decellularized group showed similar rates of early mortality (odds ratio, 0.55, 95% confidence interval, 0.21–1.41, *P* = 0.22). At a mean follow-up period of 5.89 years, no significant difference was observed between the two groups for follow-up allograft dysfunction (hazard ratio, 0.65, 95% confidence interval, 0.20–2.14, *P* = 0.48). Similarly, no difference was seen in reintervention rates (hazard ratio, 0.54, 95% confidence interval, 0.09–3.12, *P* = 0.49) nor endocarditis (hazard ratio, 0.30, 95% confidence interval, 0.07–1.35, *P* = 0.12) at a mean follow-up of 4.85 and 5.75 years, respectively.

**Conclusions:**

Decellularized and cryopreserved pulmonary allografts are associated with similar postoperative outcomes for RVOT reconstruction during the Ross procedure. Larger propensity-matched and randomized control trials are necessary to elucidate the efficacy of decellularized allografts compared to cryopreserved allografts in the setting of the Ross.

**Supplementary Information:**

The online version contains supplementary material available at 10.1186/s43044-021-00226-w.

## Background

The Ross procedure continues to be a viable form of aortic valve replacement with favorable hemodynamics and long-term viability. Currently, cryopreserved pulmonary allografts are the golden standard for repair of the right ventricular outflow tract reconstruction (RVOT) during the Ross procedure, but their use has been associated with increased risk of reoperation and immunological responses. Conversely, the decellularized grafts have been proposed to achieve the favorable hemodynamics associated with a pulmonary allograft, while reducing the risk of premature graft stenosis. While previous isolated observational studies have reported the clinical outcomes of decellularized and cryopreserved allografts use in the Ross procedure, consensus over the optimal strategy is still lacking. Herein, we perform a pairwise meta-analysis to compare the short-term and long-term outcomes of decellularized versus cryopreserved pulmonary allografts for RVOT reconstruction during the Ross procedure.

## Introduction

For younger patients undergoing aortic valve replacement, the Ross procedure offers an alternative to artificial conduits and has been shown to have favorable hemodynamics and long-term viability [1]. Additionally, the use of a living valve substitute bypasses the risk of life-long coagulation therapy [2] and increased risk of structural valve deterioration [3] associated with mechanical and bioprosthetic valves, respectively. Following the insertion of the pulmonary autograft in the aortic position during the Ross procedure, attention is directed toward the repair of the right ventricular outflow tract (RVOT). While cryopreserved pulmonary allografts are the current golden standard for RVOT reconstruction during the Ross procedure [4], their use has been associated with increased risk of reoperation due to calcifications and damage to the graft by host immunological response [5]. Alternatively, the use of decellularization techniques has been proposed as a means of providing the optimal hemodynamic associated with a pulmonary allograft, while reducing the risk of premature graft stenosis [6, 7]. Previously, the clinical outcomes of decellularized and cryopreserved allografts use in the Ross procedure have been reported through isolated observational studies [8–11], but consensus over the optimal strategy is still lacking. Herein, we perform a pairwise meta-analysis to compare the short-term and long-term outcomes of decellularized versus cryopreserved pulmonary allografts for RVOT reconstruction during the Ross procedure.

## Methods

### Comprehensive search strategy

This study was registered on the PROSPERO database of systematic reviews (ID: CRD42021247939) and followed the Meta-analysis of observational studies in epidemiology (MOOSE) proposal (Additional file 1: Fig. S1) [12]. Institutional ethical approval was not required as no individual patient data were utilized during this meta-analysis. A comprehensive literature search was performed on May 1, 2021, to find trials and observational cohort investigations comparing the use of decellularized and cryopreserved pulmonary allografts for RVOT reconstruction in patients undergoing the Ross procedure. The search strategy was performed with no restrictions on PubMed (National Institute of Health), Ovid EMBASE, and Scopus and utilized the terms: ‘Ross,’ ‘Ross-Yacoub,’ ‘decellularized,’ ‘cryopreserved,’ ‘allograft,’ and ‘homograft.’ Recently published articles making comparisons between allograft use during the Ross procedure were hand-searched and screened. A full outline of the search strategies for PubMed, EMBASE and Scopus is collated in Additional file 1: Table S1.

### Study screening

**Fig. 1:**
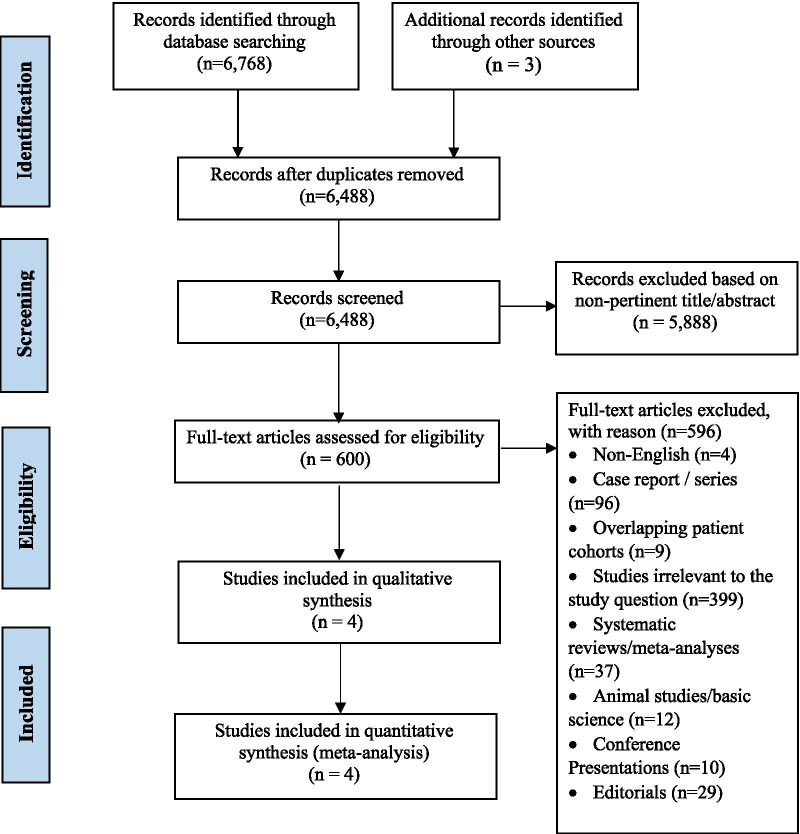
Preferred reporting items for systematic reviews and meta-analysis (PRISMA) flowchart of our analysis

The literature search yielded 6768 results (Fig. 1). After deduplication, two investigators (S.A. and R.P.) individually reviewed 6488 titles and abstracts of remaining studies using pre-defined criteria. Any discrepancies were resolved by the senior author (K.H.L.). Articles were eligible for inclusion if they were published in the English language and directly compared outcomes of patients undergoing RVOT reconstruction during the Ross procedure using either a decellularized or cryopreserved pulmonary allograft. Studies were excluded if they were abstracts, posters, animal studies, case series, commentaries, presentations, or systematic reviews. Studies reporting outcomes for single-arm interventions were also excluded from analysis.

Next, full texts of remaining studies assessed for eligibility. Additionally, any relevant citations for included articles were screened for inclusion. For overlapping patient cohorts, only the study with the larger sample size was included. Full texts were evaluated by two authors (K.S.V. and D.M.M.) and followed by confirmation of accuracy by a third investigator (A.A.).

### Quality assessment

Using the Newcastle–Ottawa Scale for observational studies, two authors (S.A. and R.P.) independently reviewed included studies for quality; discrepancies were resolved by discussion to reach consensus (Additional file 1: Table S2) [13].

### Data collection

Two authors (D.O.R. and S.A.S.) recorded data for included studies independently, and their collected data were then verified by a third investigator for accuracy (P.J.F.). Whenever reported by an included study, propensity-matched data were extracted for our analysis.

When available, information was collected regarding: study information [cohort size, period of study, institution of study, and country of study] (Table 1), patient baseline characteristics [age, gender, body mass index (BMI), body surface area (BSA), New York Heart Association (NYHA) Class III/IV, presenting etiology (congenital, degenerative, endocarditis, rheumatic, prosthetic valve dysfunction)], and comorbidities [high blood pressure, angina, atrial fibrillation, coronary artery disease, smoking status, peripheral artery disease, peripheral vascular disease, cerebrovascular disease, congenital disease, chronic obstructive pulmonary disease, renal insufficiency, prior cardiothoracic operations, left ventricular ejection fraction, and diabetes mellitus] (Additional file 1: Table S3). Additionally, we recorded data pertaining to operative details [operative technique (root replacement, inclusion, subcoronary implantation, redo allograft), allograft mean diameter (mm), ABO blood type mismatch, RVOT augmentation] (Additional file 1: Table S4).

**Table 1:** Summary of included studies

References	Study period	Hospital	Country	Types of study	Decellularized allograft	Cryopreserved allograft
Bechtel [8]	2000–2002	University of Luebeck	Germany	Retrospective	23	49
Brown [11]	2000–2005	Multicenter	USA	Retrospective	193	665
Chauvette [9]	2011–2019	Multicenter, Canadian Ross Registry	Canada	Retrospective	466	31
Etnel [10]	1995–2017	Santa Case de Curitiba	Brazil	Retrospective/PSM	130	130

PSM: Propensity-score matching

### Statistical analysis

**Fig. 2:**
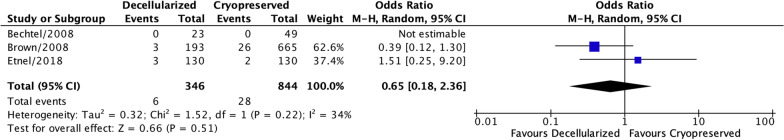
Forest plot comparing early mortality in decellularized allografts versus cryopreserved allografts for patients undergoing right ventricular outflow tract reconstruction during the Ross procedure. *MH* Mantel–Haenszel, *CI* confidence interval

**Fig. 3:**
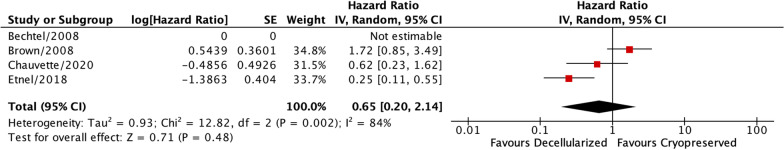
Forest plot comparing follow-up allograft dysfunction in decellularized allografts versus cryopreserved allografts for patients undergoing right ventricular outflow tract reconstruction during the Ross procedure. *IV* inverse variance, *CI* confidence interval

**Fig. 4:**
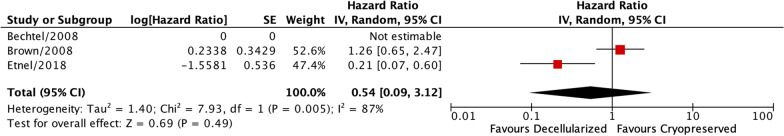
Forest plot comparing reinterventions in decellularized allografts versus cryopreserved allografts for patients undergoing right ventricular outflow tract reconstruction during the Ross procedure. *IV* inverse variance, *CI* confidence interval

**Fig. 5:**
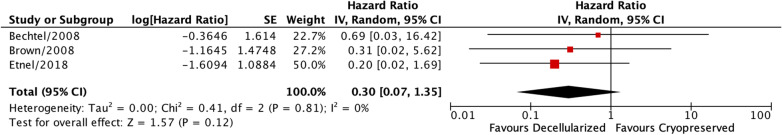
Forest plot comparing follow-up endocarditis in decellularized allografts versus cryopreserved allografts for patients undergoing right ventricular outflow tract reconstruction during the Ross procedure. *IV* inverse variance, *CI* confidence interval

All conducted statistical analyses were performed in Review Manager (RevMan 5.4.1, The Cochrane Collaboration, 2020) [14] using the Mantel–Haenszel method. For all analyses, a 2-tailed *P* value < 0.05 was deemed statistically significant. Using the random-effects model, we pooled early outcomes as odds ratio (OR) and their 95% confidence intervals (CIs). All of our follow-up outcomes (based on longest available follow-up) were pooled as a natural logarithm of the hazard ratio (HR) using the generic inverse variance method to account for any heterogeneity between follow-up durations for each intervention group. We recorded heterogeneity as low (*I*^2^ = 0–25%), moderate (*I*^2^ = 25–50%), or high (*I*^2^ > 50%) using Higgins’ and Thompson’s *I*^2^ statistic [15] (Figs. 2, 3, 4 and 5). We assessed publication bias using a funnel plot (Additional file 1: Figs. S2.1–S2.4).

### Outcomes and definitions

Early mortality and follow-up allograft dysfunction were our primary outcomes. Our secondary outcomes were reintervention rates and follow-up endocarditis. Outcome definitions in this analysis corresponded to the definitions reported in our included studies (Additional file 1: Table S5).

## Results

The comprehensive search strategy retrieved 6768 studies, four of which were found to be eligible for analysis (Fig. 1). All studies were retrospective cohort investigations. One of the studies was propensity-matched. Of the included studies, two were multicenter studies. One investigation originated from Germany, the USA, Brazil, and Canada, respectively.

In total, 1687 patients undergoing RVOT reconstruction during the Ross procedure were included; of these, 812 patients received decellularized pulmonary allografts, while 875 patients received cryopreserved pulmonary allografts. Study characteristics are summarized in Table 1. In all studies, patients directly underwent a Ross procedure, in which their native aortic valve was excised, followed by harvesting of the pulmonary autograft taken from their RVOT. Autografts were implanted in the aortic valve position using either the full root replacement or inclusion technique. Two studies reported the use of blood cardioplegia for myocardial protection [8, 10]. This was followed by the implant of either a decellularized or cryopreserved pulmonary allograft to repair the RVOT. Distal suture lines were made with running 5–0 polypropylene sutures, while proximal suture lines were made with 4–0 continuous polypropylene sutures.

### Baseline characteristics

Total number of patients included in each study ranged from 72 to 858, while the mean age ranged from 28 to 47 years (28–47 years in the decellularized group, and 28.3–46.4 years in the cryopreserved group). Patients in both groups did not significantly differ in age (MD, − 0.92, 95% CI − 7.15 to 5.31, *P* = 0.77) (Additional file 1: Fig. S3.1). Female sex ranged from 17.4 to 35.0% (17.4–29.0% in the decellularized group and 24.0–35.0% in the cryopreserved group). There was no significant difference between decellularized and cryopreserved allografts for percentage of female patients (26.8% vs. 25.5%, respectively, *P* = 0.95) (Additional file 1: Fig. S3.2). The proportion of patients with previous cardiac surgeries ranged from 7.5 to 29% (11–20.8% in the decellularized group and 7.5–29% in the cryopreserved group). Pooled analysis showed no significant difference between the two groups for percentage of patients with previous cardiac surgeries (15.0% vs. 10.5%, respectively, *P* = 0.76) (Additional file 1: Fig. S3.3). Preoperative homograft diameter (mm) ranged from 24.6 to 29.1 mm (24.6–29.1 mm in the decellularized group and 25.1–28.2 mm in the cryopreserved group). Preoperative homograft diameter did not differ significantly between the two groups (MD, 0.08, 95% CI − 0.67–0.83, *P* = 0.83) (Additional file 1: Fig. S3.4). Hypertension ranged from 12.3 to 32% (15.4–32% in the decellularized group and 12.3–23% in the cryopreserved group). There was no significant difference between decellularized and cryopreserved allografts for percentage of patients with hypertension (28.4% vs. 14.3%, respectively, *P* = 0.21) (Additional file 1: Fig. S3.5). The proportion of patients with smoking/tobacco use ranged from 8.5 to 32% (9.2–26% in the decellularized group and 8.5–32% in the cryopreserved group). Pooled analysis showed no significant difference between the two groups for smoking/tobacco use (22.3% vs. 13%, respectively, *P* = 0.67) (Additional file 1: Fig. S3.6). Chronic obstructive pulmonary disease ranged from 0 to 5% (0.8–5% in the decellularized group and 0–3% in the cryopreserved group). No significant difference was seen between the decellularized and cryopreserved groups for chronic obstructive pulmonary disease (4% vs. 0.6%, respectively, *P* = 0.47) (Additional file 1: Fig. S3.7).

### Primary outcomes

#### Early mortality

There was no significant difference between decellularized and cryopreserved allografts for early mortality (OR, 0.65, 95% CI 0.18–2.36, *P* = 0.51). *I*^2^ was 34%, suggesting moderate heterogeneity (Fig. 2).

#### Follow-up allograft dysfunction

Weighted mean follow-up duration for allograft dysfunction was 5.89 years. There was no statistically significant difference between the two groups for follow-up allograft dysfunction (HR, 0.65, 95% CI 0.20–2.14, *P* = 0.48). Heterogeneity was high between the included studies (*I*^2^ = 84%) (Fig. 3).

### Secondary outcomes

#### Reintervention

The weighted mean follow-up for reintervention was 4.85 years. There was no significant difference between decellularized and cryopreserved allografts for reintervention (HR, 0.54, 95% CI 0.09–3.12, *P* = 0.49). The studies were highly heterogeneous (*I*^2^ = 87%) (Fig. 4).

#### Follow-up endocarditis

The weighted mean follow-up duration for endocarditis was 5.75 years; no statistically significant difference in follow-up endocarditis was found between the two groups (HR, 0.30, 95% CI 0.07–1.35, *P* = 0.12). Low heterogeneity between the studies was found (*I*^2^ = 0%) (Fig. 5).

## Discussion

Despite its technical difficulty, the Ross procedure is currently considered a viable alternative to mechanical and bioprosthetic aortic valve replacements, particularly for children and young adults due to its favorable long-term hemodynamics [16]. However, while the pulmonary autograft inserted in the aortic position provides long-term viability, the operation has been criticized due to its association with dysfunction of the pulmonary allograft, including premature stenosis [5] and elevated gradients [17]. Cryopreserved grafts have traditionally been the conduit of choice for RVOT reconstruction during the Ross procedure, but their use has been shown to trigger an increased immune response from host cells. Host hypersensitivity has been proposed as a major cause behind late allograft dysfunction, with risk factors including younger donor age, shorter periods of cryopreservation, and smaller conduit size [18]. To combat this, several centers [19, 20] have reported varying success with decellularization techniques, which are believed to provide reduced antigenicity and long-term host compatibility. However, the superiority of decellularized allografts over cryopreserved for RVOT reconstruction in the setting of the Ross has yet to be clearly established with surgeons depending on isolated observational evidence to guide their decision.

In this meta-analysis of 4 studies (1687 patients), we found no significant differences in any explored outcomes between decellularized and cryopreserved allografts for repair of the RVOT in patient undergoing the Ross procedure, including early mortality, follow-up allograft dysfunction, reintervention rates, and follow-up endocarditis. To our knowledge, this is the first meta-analysis directly comparing the clinical outcomes of patients receiving decellularized or cryopreserved allografts for RVOT reconstruction during the Ross procedure. Previously, Waqanivavalagi and colleagues [21] sought to comprehensively assess the outcomes of decellularized versus cryopreserved heart valves in patients undergoing RVOT reconstruction. Their meta-analysis analysis of 16 studies including 4143 patients found no difference in postoperative mortality (RR, 0.94, 95%CI 0.60–1.47, *P* = 0.77), but significantly lower reoperation rates (RR, 0.55, 95%CI 0.36–0.84, *P* = 0.0057) for the decellularized group. However, their pooled sample size was comprised of patients with different etiologies requiring outflow tract reconstruction, which may have confounded the mechanisms by which allograft dysfunction arose. The exclusive use of Ross patients in our analysis may be particularly useful for comparing the functionality of decellularized and cryopreserved conduits because the grafts can be precisely sized before sutured into the bed of the transplanted autograft, while preserving the pulmonary tree of the patient [7].

In line with previous literature, our analysis supports the non-inferiority of decellularized pulmonary allograft durability compared to cryopreserved allografts, highlighted by the similar rates of allograft dysfunction and reintervention rates over time between the two groups. Interestingly, in a study of 52 patients receiving outflow tract reconstruction, decellularized allografts demonstrated decreased regurgitation rates at a short-term follow-up of 19 ± 13 months when compared to cryopreserved allografts [22]. However, in a combined series of 82 patients undergoing either Ross or Rastelli procedures at the same institution, Konuma et al. [23] reported no difference in early or late allograft insufficiency or stenosis when assessed at a long-term follow-up of 46 ± 14 months. This may suggest a biphasic short-term benefit to decellularized allografts that is lost over time. Our analysis at a long-term follow-up of 5.89 and 4.85 years supports these findings with no difference being shown between the two groups for allograft dysfunction and re-intervention, respectively. Additionally, previous reports have reported similar rates of endocarditis between decellularized and cryopreserved allografts during the Ross procedure [24]. Our analysis is in line with this, showing an overall endocarditis risk of 0.7% in our cohort at a weight mean follow-up of 5.75 years with no significant difference between the decellularized and cryopreserved groups. The lower risk of endocarditis following the use of pulmonary allografts compared to other conduits for repairing RVOT continues to be a benefit for both decellularized and cryopreserved grafts [24].

The results of our meta-analysis must be interpreted in the context of several limitations. Firstly, moderate to high heterogeneity was present in three of our pooled outcomes. To help ensure we did not over-estimate the association between type of pulmonary allograft and clinical outcome, all our analyses were performed using the random-effects model. Secondly, there was a paucity of literature making comparisons between the use of these two conduits for repairing the RVOT in the context of the Ross, with our final analysis consisting of four eligible studies. This limited our ability to pool further baseline characteristics and postoperative outcomes. Further, while our funnel plots showed no evidence of publication bias, having a limited number of eligible studies may have hindered the ability for the funnel plots to show asymmetry. Due to overlapping cohorts, we had to exclude several comparative studies [25–27] from our pooled-analysis, which may have decreased our statistical power and limited our ability to explore additional outcomes, such as long-term mortality and hemodynamics. We also could not assess the immunological profiles and pro-inflammatory markers in the included patients, which may have provided insight into the mechanisms causing allograft-related dysfunction. Finally, there was heterogeneity in the definitions of outcomes between studies, as some did not report their endpoints using standardized definitions [28].

The Ross procedure continues to show its utility in younger patients requiring aortic valve replacement, showing low rates of reoperation in the first two decades following intervention, regardless of whether a decellularized or cryopreserved allograft was used to repair the RVOT [29, 30]. Despite this, the operation’s use has dramatically decreased in the past decade due to operative complexity and fear of long-term durability [31]. This trend coupled with the advent of valve-in-valve technology [32, 33] for patients requiring repeat aortic valve interventions greatly diminishes the hope of definitive randomized trials comparing the use of decellularized and cryopreserved allografts for RVOT reconstruction during the Ross procedure. Therefore, in the absence of high-evidence trials and larger, multicenter propensity-matched studies, our hope is that this review will provide some guidance for heart teams as they decide the optimal treatment approach for their patients.

## Conclusions

Our results suggest that decellularized allografts have similar clinical outcomes compared to cryopreserved grafts for RVOT reconstruction during the Ross procedure, including early mortality, follow-up valve dysfunction, reintervention, and follow-up endocarditis. Larger propensity-matched studies and randomized control trials are necessary to elucidate the efficacy of decellularized conduits compared to cryopreserved allografts in the setting of the Ross.

## Supplementary Information

**Additional file 1:** Supplemental material including figures S1–S4 and tables S1–S5.

## Data Availability

All data generated or analyzed during this study are included in this published article [and its Additional file 1].

## Abbreviations

RVOT: Right ventricular outflow tract
MOOSE: Meta-analysis of observational studies in epidemiology
PRISMA: The preferred reporting items for systematic reviews and meta-analyses
BMI: Body mass index
BSA: Body surface area
NYHA: New York Heart Association
OR: Odds ratio
CI: Confidence intervals
HR: Hazard ratio
