# Supplementary material for: Decellularized versus cryopreserved pulmonary allografts for right ventricular outflow tract reconstruction during the Ross procedure: a meta-analysis of short- and long-term outcomes
Source: Egypt Heart J. 2021 Nov 7;73:100. doi: 10.1186/s43044-021-00226-w (PMC8572935; doi:10.1186/s43044-021-00226-w)
Supplement: Supplementary file 1 — Additional file 1: Supplemental material including figures S1–S4 and tables S1–S5. [file 43044_2021_226_MOESM1_ESM.docx]

**APPENDIX**

**Additional file 1: Figure 1.** The Meta-Analysis of Observational Studies in Epidemiology (MOOSE) Reporting checklist for our analysis.

| **Reporting Criteria** | **Reported (Yes/No)** | **Reported on Page** |
| --- | --- | --- |
| **Reporting of Background** |  |  |
| Problem definition | Yes | 3 |
| Hypothesis statement | Yes | 3 |
| Description of Study Outcome(s) | Yes | 6 |
| Type of exposure or intervention used | Yes | 3 |
| Type of study design used | Yes | 3 |
| Study population | Yes | 4 |
| **Reporting of Search Strategy** |  |  |
| Qualifications of searchers (eg, librarians  and investigators) | No |  |
| Search strategy, including time period  included in the synthesis and keywords | Yes | 4 |
| Effort to include all available studies,  including contact with authors | No |  |
| Databases and registries searched | Yes | 4 |
| Search software used, name and  version, including special features used  (eg, explosion) | No |  |
| Use of hand searching (eg, reference  lists of obtained articles) | Yes | 4 |
| List of citations located and those  excluded, including justification | Yes | 5 |
| Method for addressing articles  published in languages other than  English | Yes | 5 |
| Method of handling abstracts and  unpublished studies | Yes | 5 |
| Description of any contact with authors | No |  |
| **Reporting of Methods** |  |  |
| Description of relevance or  appropriateness of studies assembled for  assessing the hypothesis to be tested | Yes | 6 |
| Rationale for the selection and coding of  data (eg, sound clinical principles or  convenience) | Yes | 5-6 |
| Documentation of how data were  classified and coded (eg, multiple raters,  blinding, and interrater reliability) | Yes | 5-6 |
| Assessment of confounding (eg,  comparability of cases and controls in  studies where appropriate | No |  |
| Assessment of study quality, including  blinding of quality assessors;  stratification or regression on possible  predictors of study results | Yes | 7 |
| Assessment of heterogeneity | Yes | 7 |
| Description of statistical methods (eg,  complete description of fixed or random  effects models, justification of whether  the chosen models account for predictors  of study results, dose-response models,  or cumulative meta-analysis) in sufficient  detail to be replicated | Yes | 6-7 |
| Provision of appropriate tables and  graphics | Yes | 8 |
| **Reporting of Results** |  |  |
| Table giving descriptive information for  each study included | Yes | 15 |
| Results of sensitivity testing (eg,  subgroup analysis) | No |  |
| Indication of statistical uncertainty of  findings | Yes | 7 |
| **Reporting of Discussion** |  |  |
| Quantitative assessment of bias (eg,  publication bias) | Yes | 7 |
| Justification for exclusion (eg, exclusion  of non–English-language citations) | Yes | 4-5 |
| Assessment of quality of included studies | Yes | 5 |
| **Reporting of Conclusions** |  |  |
| Consideration of alternative explanations  for observed results | Yes | 11-12 |
| Generalization of the conclusions (ie,  appropriate for the data presented and  within the domain of the literature review) | Yes | 11-12 |
| Guidelines for future research | Yes | 12 |
| Disclosure of funding source | Yes | 13 |

**Additional file 1: Figure 2.1.** Funnel plot of studies reporting early mortality. OR, Odds Ratio; SE, Standard Error


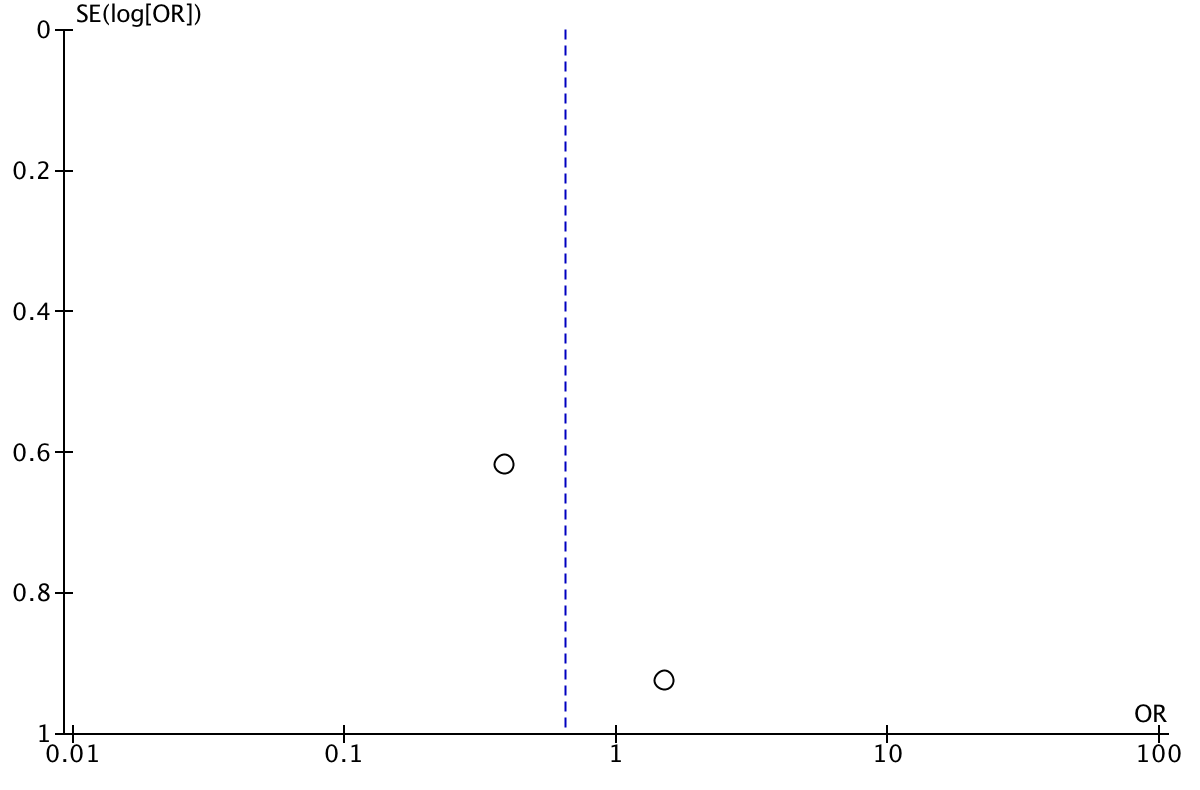


**Additional file 1: Figure 2.2.** Funnel plot of studies reporting follow-up allograft dysfunction. SE, Standard Error


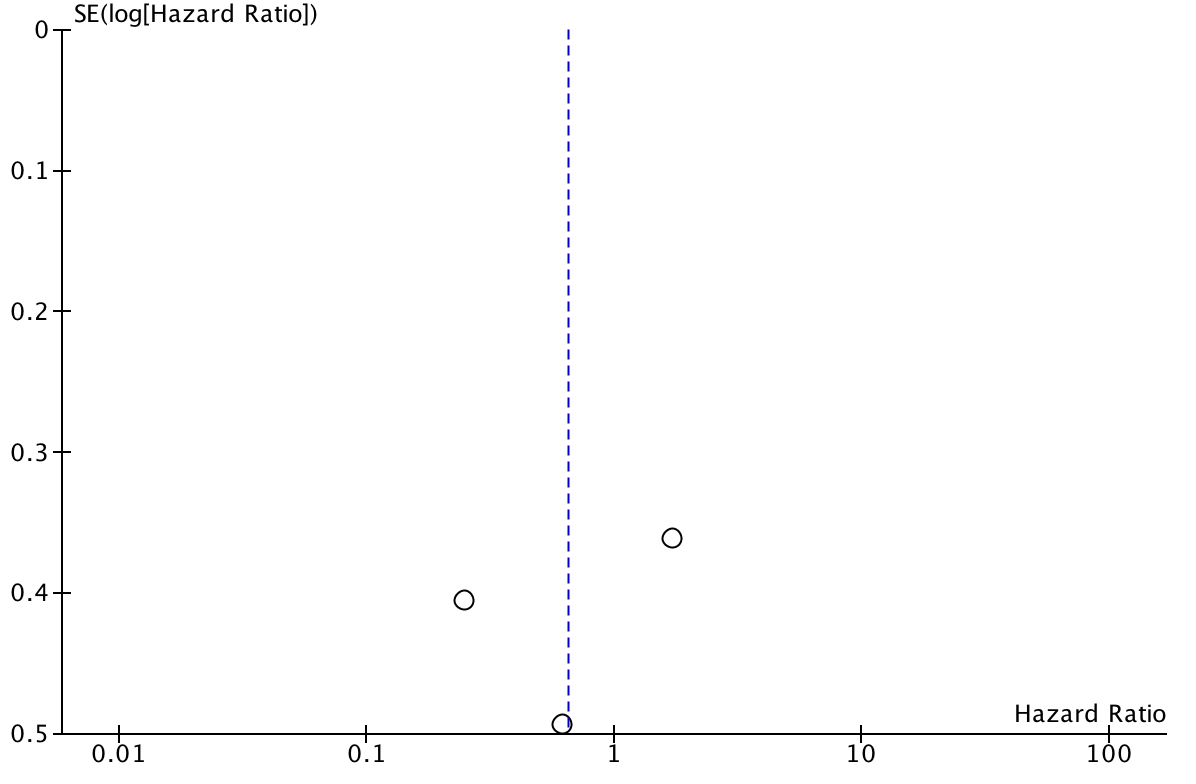


**Additional file 1: Figure 2.3.** Funnel plot of studies reporting reintervention. SE, Standard Error


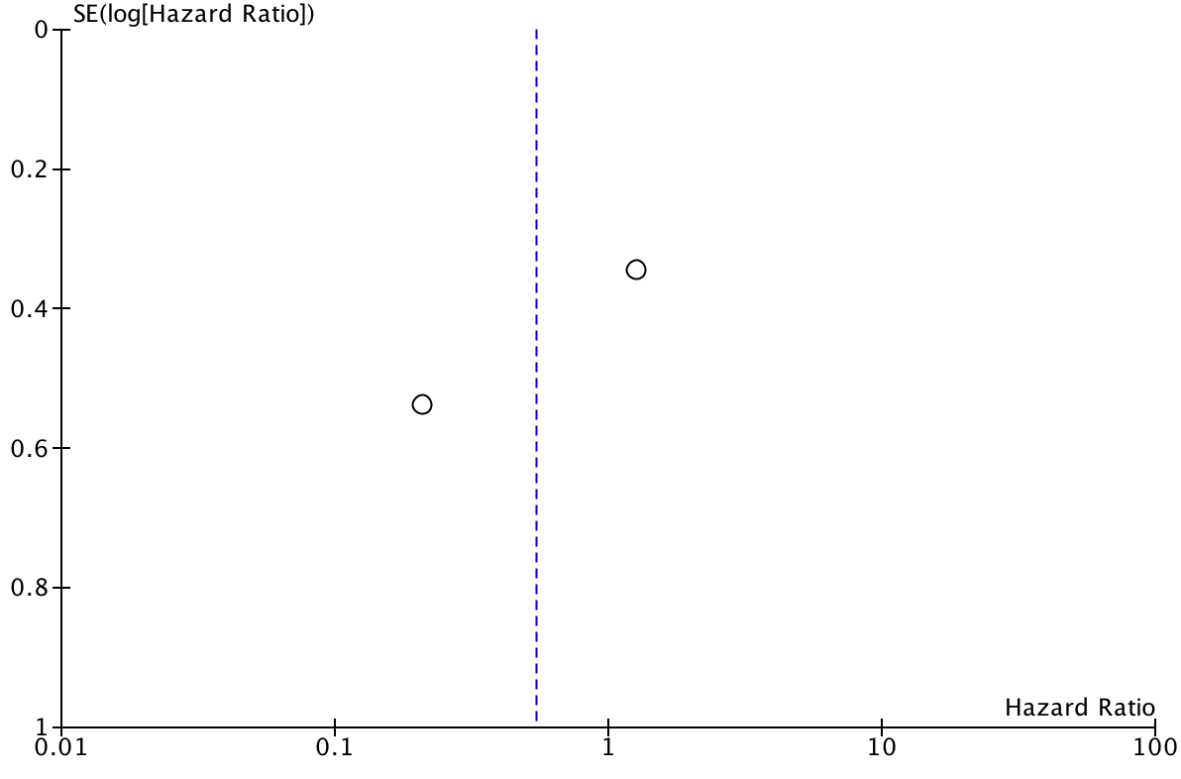


**Additional file 1: Figure 2.4.** Funnel plot of studies reporting follow-up endocarditis. SE, Standard Error


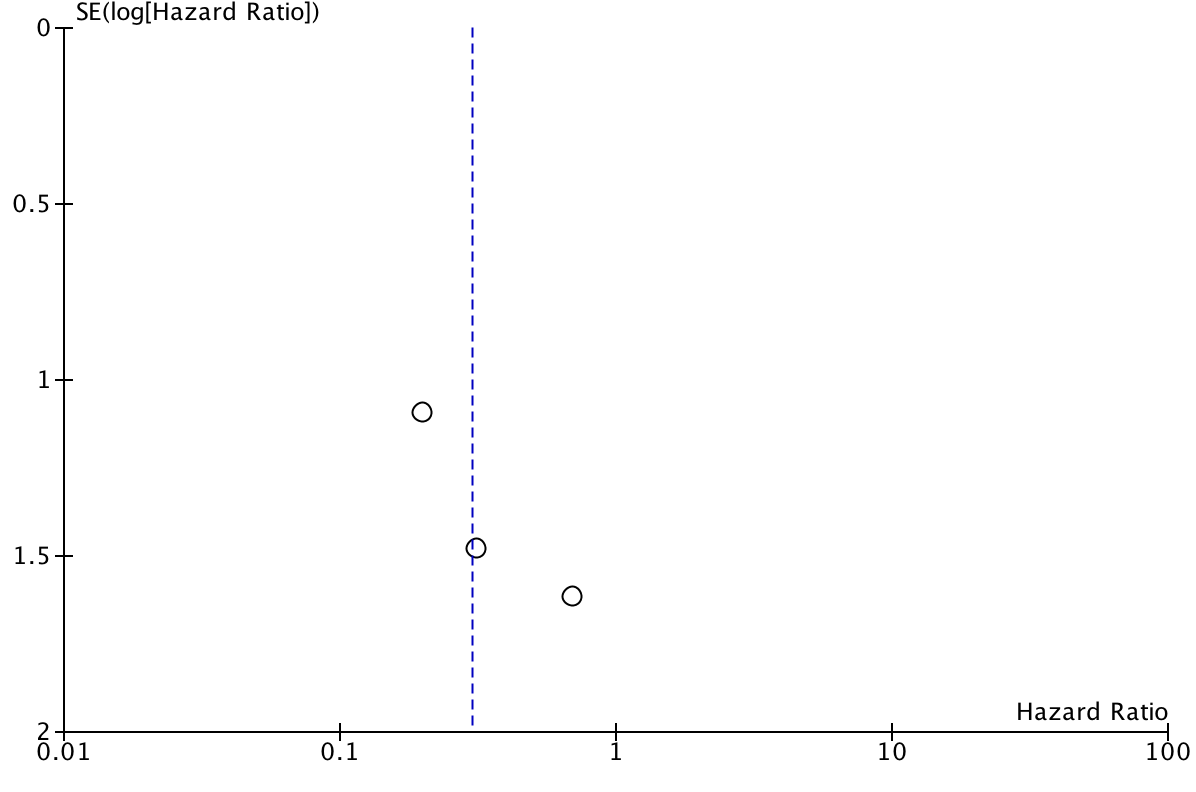


**Additional file 1: Figure 3.1** Forest plot for mean differences in age between patients undergoing right ventricular outflow tract reconstruction during the Ross procedure using a decellularized pulmonary allograft and cryopreserved pulmonary allograft. IV, Inverse Variance; CI, Confidence Interval


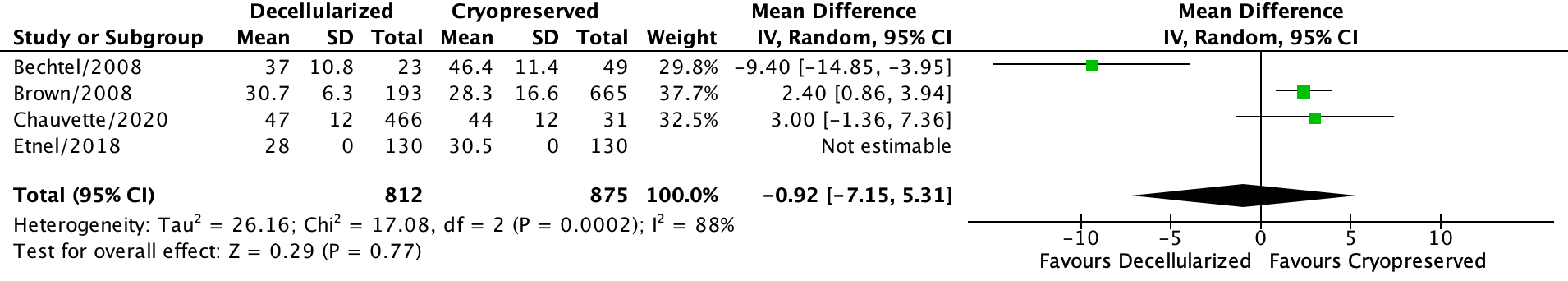


**Additional file 1: Figure 3.2** Forest plot for proportion of women undergoing right ventricular outflow tract reconstruction during the Ross procedure using a decellularized pulmonary allograft and cryopreserved pulmonary allograft. MH, Mantel-Haenszel; CI, Confidence Interval


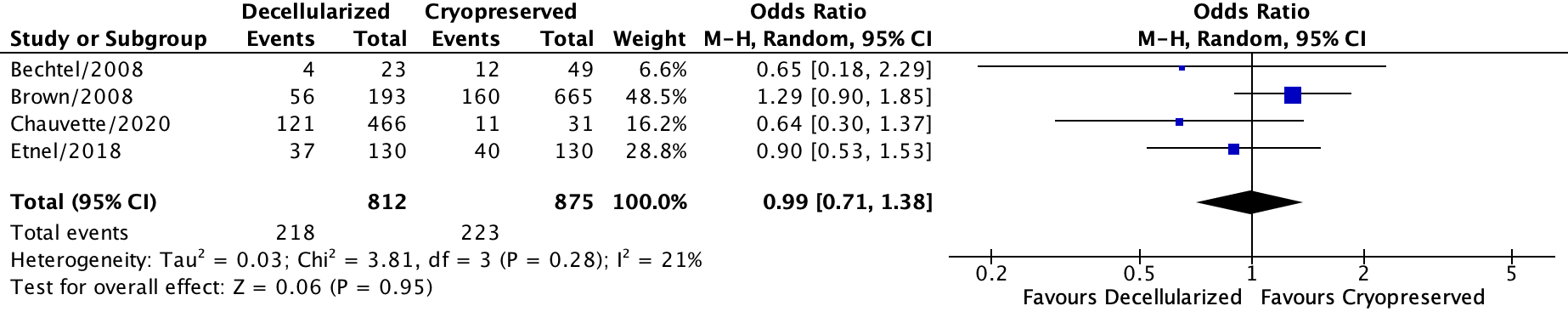


**Additional file 1: Figure 3.3** Forest plot for proportion of patients with prior cardiac surgeries undergoing right ventricular outflow tract reconstruction during the Ross procedure using a decellularized pulmonary allograft and cryopreserved pulmonary allograft. MH, Mantel-Haenszel; CI, Confidence Interval

**
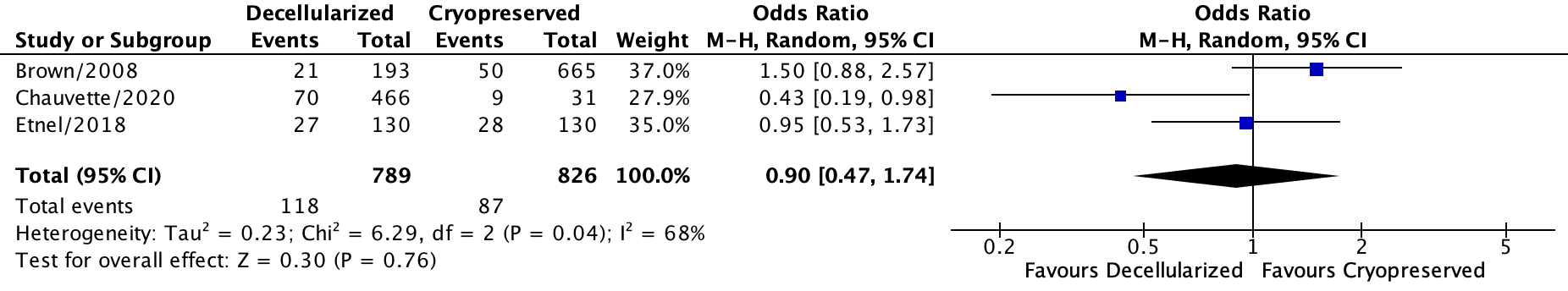
**

**Additional file 1: Figure 3.4** Forest plot for mean differences in pre-operative pulmonary homograft diameters (mm) between patients undergoing right ventricular outflow tract reconstruction during the Ross procedure using a decellularized pulmonary allograft and cryopreserved pulmonary allograft. IV, Inverse Variance; CI, Confidence Interval

**
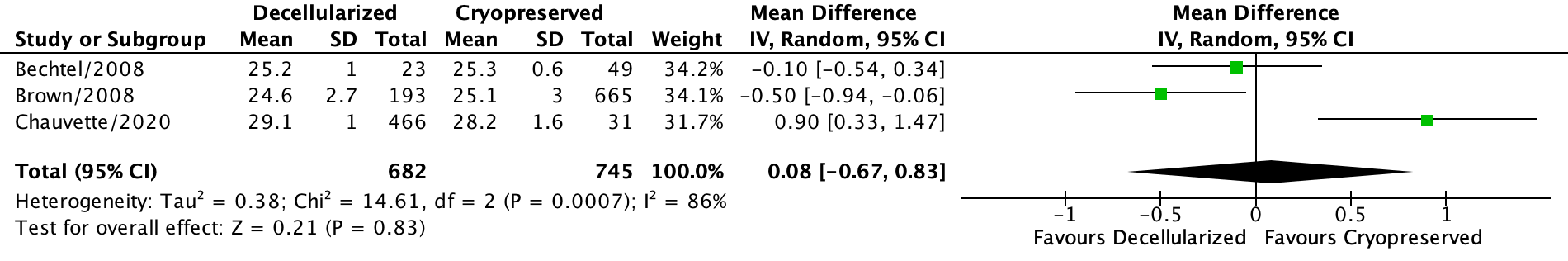
**

**Additional file 1: Figure 3.5** Forest plot for proportion of patients with hypertension undergoing right ventricular outflow tract reconstruction during the Ross procedure using a decellularized pulmonary allograft and cryopreserved pulmonary allograft. MH, Mantel-Haenszel; CI, Confidence Interval

**
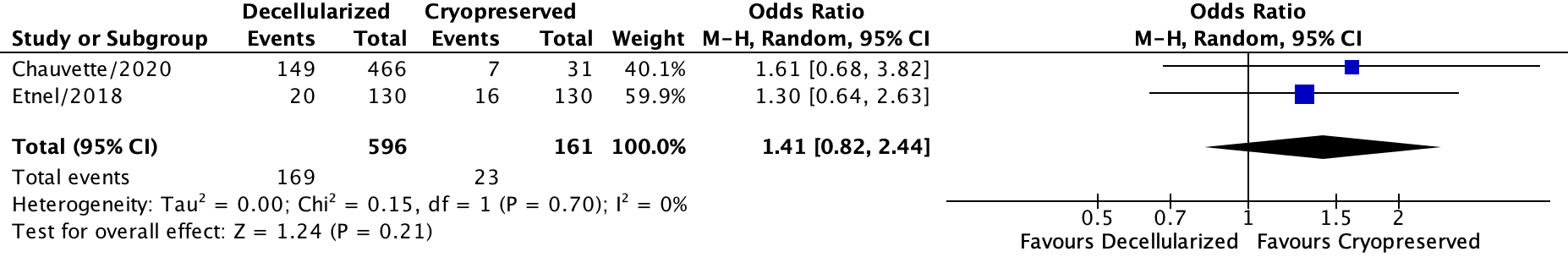
**

**Additional file 1: Figure 3.6** Forest plot for proportion of patients with smoking / tobacco use status undergoing right ventricular outflow tract reconstruction during the Ross procedure using a decellularized pulmonary allograft and cryopreserved pulmonary allograft. MH, Mantel-Haenszel; CI, Confidence Interval

**
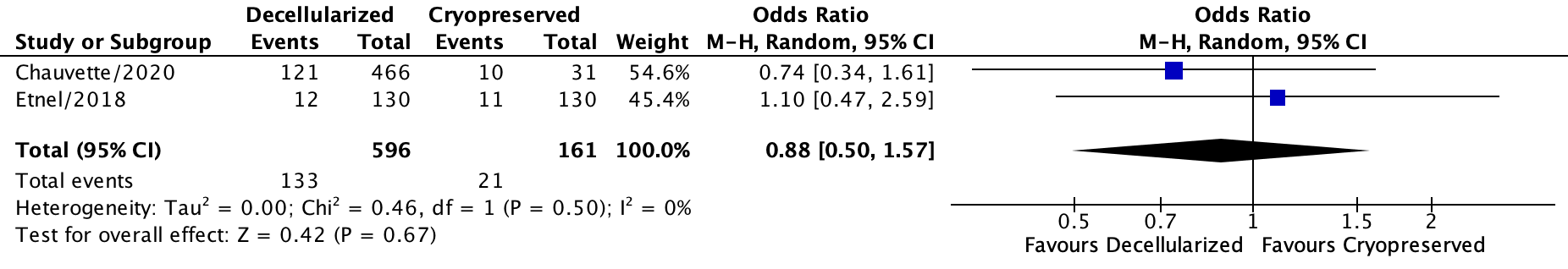
**

**Additional file 1: Figure 3.7** Forest plot for proportion of patients with chronic obstructive pulmonary lung disease undergoing right ventricular outflow tract reconstruction during the Ross procedure using a decellularized pulmonary allograft and cryopreserved pulmonary allograft. MH, Mantel-Haenszel; CI, Confidence Interval

**
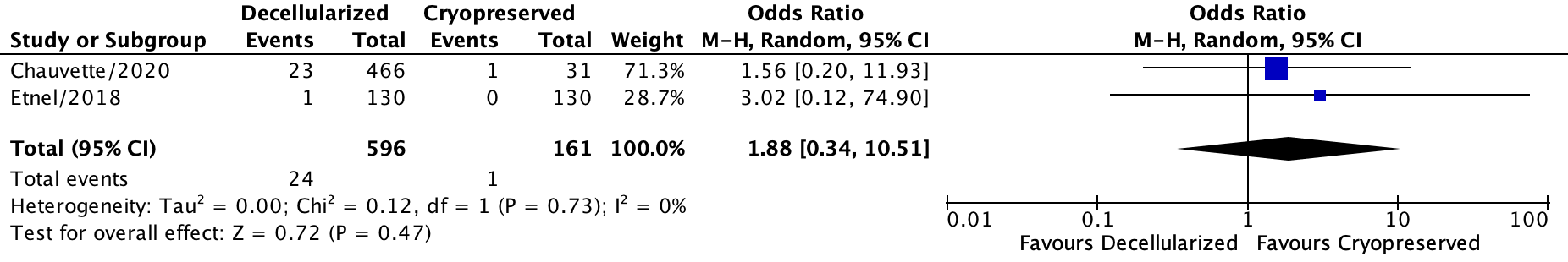
**

**Additional file 1: Table 1. Original search strategy.**

Searched on May 1, 2021 on PubMed, Ovid Embase and Scopus databases (No date, language, or article type restrictions)

(Ross procedure OR Ross-Yacoub procedure

OR pulmonary autograft OR pulmonary homograft OR pulmonary allograft

OR homograft OR autograft OR allograft)

AND ((decellularized OR de-cellularized

OR acellularization OR acellularized

OR decellularization OR decellular*

OR acellular*)

OR (cryopreserved OR cryo-preserved

OR Cryo-preservation))

**Additional file 1: Table 2. Summary of critical appraisal of included observational studies using the Newcastle Ottawa Quality Assessment Scale for Cohort Studies**

| Study/Year | Selection | Comparability | Outcome |
| --- | --- | --- | --- |
| Bechtel/2008 | **** | - | *** |
| Brown/2008 | **** | - | *** |
| Chauvette/2020 | **** | - | ** |
| Etnel/2018 | **** | ** | *** |

**Selection**

 1)        Representativeness of intervention cohort a) truly representative of the average in the community *; b) somewhat representative of the average; c) only selected group of users; d) no description of the derivation of the cohort.

2)        Selection of nonintervention cohort – a) drawn from same community as intervention cohort*; b) drawn from a different source; c) no description of the derivation of the nonexposed cohort.

3)        Ascertainment of exposure a) secure record*; b) structured interview*; c) written self-report; d) no description.

4)        Demonstration that outcome of interest was not present at start of study a) yes*; b) no.

**Comparability**

 1)       Comparability of cohorts on the basis of the design or analysis a) study controls for age, and gender*; b) study controls for any additional factor***.**

**Outcome**

 1)       Assessment of outcome a) independent blind assessment***;**b) record linkage*; c) self-report; d) no description.

2)       Was follow-up long enough for outcomes to occur a) yes*; b) no

3)       Adequacy of follow up of cohorts a) complete follow up***;**b) subjects lost to follow up unlikely to introduce bias < 20 % lost follow up***;**c) follow up rate < 80% and no description of those lost; d) no statement

**Additional file 1: Table 3.** Patient demographics, risk factors, and comorbidities.

| Study/Year | Age, mean (SD), median (IQR) [Range] | | | | Female (%) | | NYHA Functional Class III/IV (%) | |
| --- | --- | --- | --- | --- | --- | --- | --- | --- |
|  | **Decellularized** | | **Cryopreserved** | | **Decellularized** | **Cryopreserved** | **Decellularized** | **Cryopreserved** |
|  | **Mean** | **SD** | **Mean** | **SD** |  |  |  |  |
| Bechtel/2008 | 37 | 10.8 | 46.4 | 11.4 | 17.4 | 24.5 | NR | NR |
| Brown/2008 | 30.7 | 6.3 | 28.3 | 16.6 | 29 | 24 | NR | NR |
| Chauvette/2020 | 47 | 12 | 44 | 12 | 26 | 35 | NR | NR |
| Etnel/2018 | 28 | 17-42 | 30.5 | 20-39 | 28.5 | 30.8 | 36.9 | 35.2 |

| Study/Year | BSA (kg), mean (SD) | | | | BMI (kg/m^2), mean (SD) | | | | Hypertension (%) | |
| --- | --- | --- | --- | --- | --- | --- | --- | --- | --- | --- |
|  | **Decellularized** | | **Cryopreserved** | | **Decellularized** | | **Cryopreserved** | | **Decellularized** | **Cryopreserved** |
|  | **Mean** | **SD** | **Mean** | **SD** | **Mean** | **SD** | **Mean** | **SD** |  |  |
| Bechtel/2008 | 1.99 | 0.21 | 1.9 | 0.21 | NR | NR | NR | NR | NR | NR |
| Brown/2008 | NR | NR | NR | NR | NR | NR | NR | NR | NR | NR |
| Chauvette/2020 | NR | NR | NR | NR | 28 | 6 | 28 | 4 | 32 | 23 |
| Etnel/2018 | NR | NR | NR | NR | 23.55 | 20.75-26.12 | 23.52 | 21.1-25.94 | 15.4 | 12.3 |

| Study/Year | Atrial Fibrilation (%) | | Coronary Artery Disease (%) | | Smoking/Tobacco use (%) | |
| --- | --- | --- | --- | --- | --- | --- |
|  | **Decellularized** | **Cryopreserved** | **Decellularized** | **Cryopreserved** | **Decellularized** | **Cryopreserved** |
|  |  |  |  |  |  |  |
| Bechtel/2008 | NR | NR | NR | NR | NR | NR |
| Brown/2008 | NR | NR | NR | NR | NR | NR |
| Chauvette/2020 | 2 | 10 | 9 | 13 | 26 | 32 |
| Etnel/2018 | NR | NR | NR | NR | 9.2 | 8.5 |

| Study/Year | Peripheral Vascular disease (%) | | Cerebrovascular Disease (%) | | Chronic Obstructive Pulmonary Disease (%) | |
| --- | --- | --- | --- | --- | --- | --- |
|  | **Decellularized** | **Cryopreserved** | **Decellularized** | **Cryopreserved** | **Decellularized** | **Cryopreserved** |
|  |  |  |  |  |  |  |
| Bechtel/2008 | NR | NR | NR | NR | NR | NR |
| Brown/2008 | NR | NR | NR | NR | NR | NR |
| Chauvette/2020 | 0.4 | 3 | NR | NR | 5 | 3 |
| Etnel/2018 | NR | NR | 1.5 | 0.8 | 0.8 | 0 |
|  |  |  |  |  |  |  |

| Study/Year | >1 previous cardiac surgery (%) | | Angina (%) | | Prior MI (%) | | Diabetes (%) | |
| --- | --- | --- | --- | --- | --- | --- | --- | --- |
|  | **Decellularized** | **Cryopreserved** | **Decellularized** | **Cryopreserved** | **Decellularized** | **Cryopreserved** | **Decellularized** | **Cryopreserved** |
|  |  |  |  |  |  |  |  |  |
| Bechtel/2008 | NR | NR | 0 | 0 | NR | NR | NR | NR |
| Brown/2008 | 11 | 7.5 | NR | NR | NR | NR | NR | NR |
| Chauvette/2020 | 15 | 29 | NR | NR | 1 | 0 | 9 | 3 |
| Etnel/2018 | 20.8 | 21.5 | 0.8 | 0.8 | NR | NR | 0 | 0 |

| Study/Year | Preoperative Homograft Diameter (mm), mean (SD), median (IQR) [Range] | | | | Age of allograft donor | | | | Allograft donor male (%) | |
| --- | --- | --- | --- | --- | --- | --- | --- | --- | --- | --- |
|  | **Decellularized** | | **Cryopreserved** | | **Decellularized** | | **Cryopreserved** | | **Decellularized** | **Cryopreserved** |
|  | **Mean** | **SD** | **Mean** | **SD** | **Mean** | **SD** | **Mean** | **SD** |  |  |
| Bechtel/2008 | 25.2 | 1 | 25.3 | 0.6 | 48.1 | 6.9 | 48 | 9.8 | NR | NR |
| Brown/2008 | 24.6 | 2.7 | 25.1 | 3 | NR | NR | NR | NR | NR | NR |
| Chauvette/2020 | 29.1 | 1 | 28.2 | 1.6 | NR | NR | NR | NR | NR | NR |
| Etnel/2018 | NR | NR | NR | NR | 42 | 30.5-49.0 | 40 | 26.0-50.5 | 70 | 68.1 |

| Study/Year | Baseline allograft peak gradient [Pmax (mmHg)] | | | | Aortic valve hemodynamics (%) | | | | | |
| --- | --- | --- | --- | --- | --- | --- | --- | --- | --- | --- |
|  | **Decellularized** | | **Cryopreserved** | | **Decellularized** | | | **Cryopreserved** | | |
|  | **Mean** | **SD** | **Mean** | **SD** | **Stenosis** | **Regurgitation** | **Mixed** | **Stenosis** | **Regurgitation** | **Mixed** |
| Bechtel/2008 | 6.2 | 2.8 | 6.2 | 2.8 | NR | NR | NR | NR | NR | NR |
| Brown/2008 | 19 | 17.2 | 21.7 | 16.7 | NR | NR | NR | NR | NR | NR |
| Chauvette/2020 | NR | NR | NR | NR | 74 | 32 | 30 | 61 | 45 | 32 |
| Etnel/2018 | NR | NR | NR | NR | 29.2 | 34.6 | 36.2 | 30.8 | 32.3 | 36.9 |

| Study/Year | Etiology (%) | | | | | | | | | | | |
| --- | --- | --- | --- | --- | --- | --- | --- | --- | --- | --- | --- | --- |
|  | **Decellularized** | | | | | | **Cryopreserved** | | | | | |
|  | **Congenital** | **Degenerative** | **Endocarditis** | **Rheumatic** | **PV dysfunction** | **Other** | **Congenital** | **Degenerative** | **Endocarditis** | **Rheumatic** | **PV dysfunction** | **Other** |
| Bechtel/2008 | NR | NR | NR | NR | NR | NR | NR | NR | NR | NR | NR | NR |
| Brown/2008 | 64 | 0 | 0 | 0 | 0 | 36 | 55.2 | 0 | 0 | 0 | 0 | 49.77 |
| Chauvette/2020 | NR | NR | NR | NR | NR | MR | 0 | 0 | 19% | 0 | 0 | 81 |
| Etnel/2018 | 56.9 | 6.9 | 5.4 | 19.2 | 11.5 | 0 | 58.1 | 3.1 | 7 | 26.4 | 5.4 | 0 |

**Additional file 1: Table 4.** Summary of peri-operative variables of the included studies.

| Study/Year | Operative technique (%) | | | | | |
| --- | --- | --- | --- | --- | --- | --- |
|  | **Decellularized** | | | **Cryopreserved** | | |
|  | **Root replacement** | **Inclusion** | **Redo failed allograft** | **Root replacement** | **Inclusion** | **Redo failed allograft** |
| Bechtel/2008 | NR | NR | NR | NR | NR | NR |
| Brown/2008 | NR | NR | NR | NR | NR | NR |
| Chauvette/2020 | NR | NR | NR | NR | NR | NR |
| Etnel/2018 | 79.2 | 14.6 | 1.5 | 90 | 10 | 0 |

| Study/Year | Cardiopulmonary time (min), mean (SD), median (IQR) | | | | Allograft diameter (mm) (%) | | | |
| --- | --- | --- | --- | --- | --- | --- | --- | --- |
|  | **Decellularized** | | **Cryopreserved** | | **Decellularized** | | **Cryopreserved** | |
|  | **Mean** | **SD** | **Mean** | **SD** | **Mean** | **SD** | **Mean** | **SD** |
| Bechtel/2008 | 208 | 39 | 211 | 28 | NR | NR | NR | NR |
| Brown/2008 | NR | NR | NR | NR | NR | NR | NR | NR |
| Chauvette/2020 | NR | NR | NR | NR | NR | NR | NR | NR |
| Etnel/2018 | NR | NR | NR | NR | 24 | 23-26 | 25 | 23-26 |

| Study/Year | RVOT augmentation (%) | | ABO mismatch (%) | |
| --- | --- | --- | --- | --- |
|  | **Decellularized** | **Cryopreserved** | **Decellularized** | **Cryopreserved** |
|  |  |  |  |  |
| Bechtel/2008 | NR | NR | NR | NR |
| Brown/2008 | NR | NR | NR | NR |
| Chauvette/2020 | NR | NR | NR | NR |
| Etnel/2018 | 20.8 | 7.7 | 76.7 | 68.6 |

**Additional file 1: Table 5.** Definitions of outcomes in included studies

| Study/Year | Early mortality | Follow-up allograft dysfunction | Reintervention | Follow-up endocarditis |
| --- | --- | --- | --- | --- |
| Bechtel/2008 | Perioperative death | Moderate or higher regurgitation | Reoperations on autograft / allograft | Endocarditis |
| Brown/2008 | Death during the same hospitalization or within 30 days of implantation | Valve-related failure; defined as valve reintervention, valve explantation, or clinical evidence of valve-related mortality | Reintervention; according to the guidelines for reporting mortality and morbidity after cardiac valve interventions^a^ | Endocarditis: according to the guidelines for reporting mortality and morbidity after cardiac valve interventions^a^ |
| Chauvette/2020 | - | 1 or more of these criteria: peak pulmonary gradient greater than or equal to 30 mm Hg, pulmonary regurgitation >2, or pulmonary homograft reintervention | - | - |
| Etnel/2018 | Early mortality | >_Severe regurgitation and/or a peak Doppler gradient of >_36 mmHg | Reinterventions | Endocarditis |

^a^Akins CW, Miller DC, Turina MI, Kouchoukos NT, Blackstone EH, Grunkemeier GL, et al. Guidelines for reporting mortality and morbidity after cardiac valve interventions. J Thorac Cardiovasc Surg. 2008;135:732-8

**Additional file 1: Table 5.** References of Included Studies

1. Bechtel JFM, Stierle U, Sievers H-H. Fifty-two months’ mean follow up of decellularized SynerGraft-treated pulmonary valve allografts. J Heart Valve Dis. 2008 Jan;17(1):98–104; discussion 104.
2. Chauvette V, Bouhout I, Tarabzoni M, Pham M, Wong D, Whitlock R, et al. Pulmonary homograft dysfunction after the Ross procedure using decellularized homografts—a multicenter study. J Thorac Cardiovasc Surg. 2020 Jul;S0022522320321942.
3. Etnel JRG, Suss PH, Schnorr GM, Veloso M, Colatusso DF, Balbi Filho EM, et al. Fresh decellularized versus standard cryopreserved pulmonary allografts for right ventricular outflow tract reconstruction during the Ross procedure: a propensity-matched study†. Eur J Cardiothorac Surg. 2018 Sep 1;54(3):434–40.
4. Brown JW, Elkins RC, Clarke DR, Tweddell JS, Huddleston CB, Doty JR, et al. Performance of the CryoValve∗ SG human decellularized pulmonary valve in 342 patients relative to the conventional CryoValve at a mean follow-up of four years. J Thorac Cardiovasc Surg. 2010 Feb;139(2):339–48.
